# Context based mixture model for cell phase identification in automated fluorescence microscopy

**DOI:** 10.1186/1471-2105-8-32

**Published:** 2007-01-30

**Authors:** Meng Wang, Xiaobo Zhou, Randy W King, Stephen TC Wong

**Affiliations:** 1 Center for Bioinformatics, Harvard Center for Neurodegeneration and Repair, Harvard Medical School, 3rd floor, 1249 Boylston, Boston, MA 02215, USA; 2 Functional and Molecular Imaging Center, Department of Radiology, Brigham and Women's Hospital, One Brigham Circle, 1620 Tremont Street, Boston, MA 02121, USA; 3 Department of Cell Biology, Harvard Medical School, Boston, MA 02115, USA

## Abstract

**Background:**

Automated identification of cell cycle phases of individual live cells in a large population captured via automated fluorescence microscopy technique is important for cancer drug discovery and cell cycle studies. Time-lapse fluorescence microscopy images provide an important method to study the cell cycle process under different conditions of perturbation. Existing methods are limited in dealing with such time-lapse data sets while manual analysis is not feasible. This paper presents statistical data analysis and statistical pattern recognition to perform this task.

**Results:**

The data is generated from Hela H2B GFP cells imaged during a 2-day period with images acquired 15 minutes apart using an automated time-lapse fluorescence microscopy. The patterns are described with four kinds of features, including twelve general features, Haralick texture features, Zernike moment features, and wavelet features. To generate a new set of features with more discriminate power, the commonly used feature reduction techniques are used, which include Principle Component Analysis (PCA), Linear Discriminant Analysis (LDA), Maximum Margin Criterion (MMC), Stepwise Discriminate Analysis based Feature Selection (SDAFS), and Genetic Algorithm based Feature Selection (GAFS). Then, we propose a Context Based Mixture Model (CBMM) for dealing with the time-series cell sequence information and compare it to other traditional classifiers: Support Vector Machine (SVM), Neural Network (NN), and K-Nearest Neighbor (KNN). Being a standard practice in machine learning, we systematically compare the performance of a number of common feature reduction techniques and classifiers to select an optimal combination of a feature reduction technique and a classifier. A cellular database containing 100 manually labelled subsequence is built for evaluating the performance of the classifiers. The generalization error is estimated using the cross validation technique. The experimental results show that CBMM outperforms all other classifies in identifying prophase and has the best overall performance.

**Conclusion:**

The application of feature reduction techniques can improve the prediction accuracy significantly. CBMM can effectively utilize the contextual information and has the best overall performance when combined with any of the previously mentioned feature reduction techniques.

## Background

Quantitating the changes in cell cycle timing before and after drug treatment is useful for effective drug discovery research. Knowledge of the cell cycle progression, e.g., interphase, prophase, metaphase, and anaphase, is important to improving our understanding of the effects of various drugs on cancer cells [1-4]. Cell cycle progress can be identified by measuring changes in the nucleus as a function of time. Automated time-lapse fluorescence microscopy imaging provides an effective method to observe and study nuclei dynamically and is an important quantitative technique in the fields of cell biology and systems biology [2-4]. Nevertheless, the vast amount and complexity of image data acquired from automated microscopy renders manual analysis unreasonably time-consuming. Accurate automatic classification of cell nuclei into interphase, prophase, metaphase, or anaphase, is an unresolved issue in cell biology studies using fluorescence microscopy. Murphy et al. [5-9] have proposed different feature extraction, feature reduction, and classification algorithms for a similar problem of classification of subcellular location patterns in fluorescence microscope images. Methods have also been proposed to identify the cell cycle phase recognition. Gallardo et al. [10] used Hidden Markov Models (HMMs) to classify the feature vector sequences that are extracted from the segmented, potential mitotic cells. Chen et al. [11] proposed an automated system to segment, classify, and track individuals in live cell population, in which the KNN classifier with a set of seven features was used. A novel hybrid fragments merging method based on watershed segmentation and HMMs is also proposed for cell phase identification [1,2].

**Figure 1:**
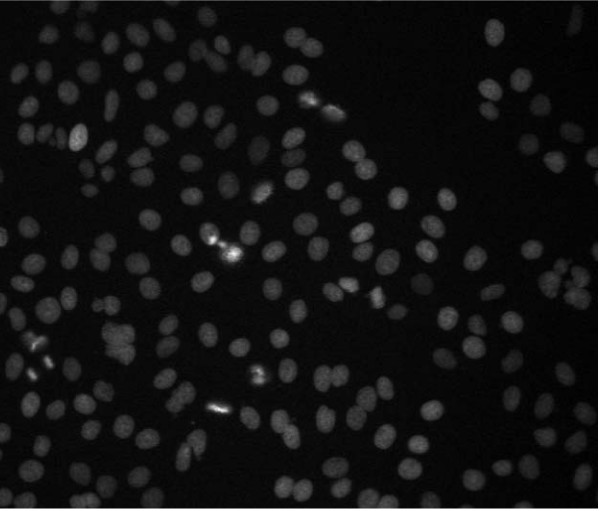
**A gray level image of a population of cells showing only nuclei channel**. The image shows the nuclei after image enhancements.

In this work, an automated analytical system [1,2] is used to acquire images, track cell nuclei and generate features of each cell nucleus in a population of thousands of cells. In these specific time-lapse fluorescence microscopy images, nuclei are bright objects protruding out from a relatively uniform dark background; see an example in Figure 1. The cell nuclei are segmented from the acquired images and represented by a group of features for phase identification. To extract features from these time-lapse fluorescence images, four operating steps are conducted [1,2]: image preprocessing, cell nuclei segmentation, fragment merging, and cell nuclei tracking. After that, 145 features are extracted from each cell nucleus. But there are many noisy and functionally redundant features. Thus it is necessary to remove the noisy, irrelevant, and redundant features with feature reduction techniques. Many feature reduction methods have been proposed to improve the efficiency and effectiveness of cell phase identification [1,6,7,12,13].

Feature reduction techniques can be generally classified into feature extraction and feature selection approaches [13,15-19]. Commonly used feature extraction algorithms, such as PCA [16,17], Linear Discriminant Analysis (LDA) [17,18], and Maximum Margin Criterion (MMC) [19] are investigated in this work. In PCA, the linear projections of the greatest variance from the top eigenvectors of the covariance matrix are computed, which works well when the data lies close to a flat manifold. LDA is one of the most commonly used supervised feature extraction algorithms. It is used to locate a lower dimensional space that best discriminates the samples from different classes. LDA explicitly utilizes the label information of the samples and thus is suitable for classification problems. However, it often suffers from small sample size when dealing with the high dimensional image data. Moreover, while LDA is guaranteed to find the best directions when each class has a Gaussian density with a common covariance matrix, it may fail if the class densities are more generalized. To solve the limitations of LDA, MCC, a supervised approach, has recently been proposed. The computation complexity of MMC is lower than LDA. In addition, the SDAFS is proposed as the best approach in feature selection for cell phase identification [7]. Other approaches, such as MIFS [15,20], GAFS [21], and T-test based Feature Selection (TFS) [22], are also evaluated. It is a NP-hard problem to determine the optimal feature subset for MIFS by global search. So we adopt a greedy searching algorithm, in which the features with the highest average mutual information are selected. Genetic algorithm [17,21] is a classical random optimization method, which mimics the evolutionary process of survival of the fittest. A T-test is used to generate the initial individual feature subset, which belongs to the "Population" in the GA algorithm. To classify cell nuclei, some researchers have used KNN [11,17], BPNN [8], and SVM [23,24] to classify cell nuclei. Though acceptable results have been reported, both of them ignored the contextual information of time-lapse microscopy. To utilize the contextual information, we propose a Context Based Mixture Model. Finally, as a standard practice in machine learning, a systematic comparison is conducted to select an optimal combination of a feature reduction technique and a classifier.

Feature reduction techniques can effectively improve the prediction accuracy, and more features do not necessarily guarantee better performance. Our finding indicates that CBMM outperforms SVM, BPNN, and KNN in identifying prophase and achieves the best overall performance.

## Results

### Key Steps

The experiments consist of the following six steps.

Step 1. Image processing: Include image pre-processing, thresholding, fragment merging, and tracking. The cell nuclei are segmented from the background and tracked as cell sequences, refer to [1,2,25-27] for a detailed description.

Step 2. Feature generation: Generate 145 features for each cell nucleus in all tracked sequences.

Step 3. Data labeling and splitting: Label 100 cell sequences manually as interphase, prophase, metaphase, and anaphase.

Step 4. Feature reduction: Use the six different approaches introduced in this paper to reduce the dimension of vector space.

Step 5. Classifier training: Train the classifiers using reduced training data, the four classifiers are trained using the reduced training data obtained from step 4.

Step 6. Phase identification: Identify cell cycle phases of the cells from the reduced testing data.

### Materials

The data is generated from HeLa H2B GFP cells imaged during a 2-day period with images acquired 15 minutes apart using an automated time-lapse fluorescence microscopy. H2B GFP is a recombinant protein that localizes to DNA and is fluorescent. Each image has a resolution of 672*512 pixels. To get more reliable training data from each tracked cell sequence, we select the frames where the cell is in mitosis, thus including interphase, prophase, metaphase, and anaphase. In addition, the subsequence is supposed to start from a cell in interphase and end with a cell in anaphase. There are totally 100 manually labeled subsequences. A typical 200-frames sample of digital microscope images contains at least 18,000 interphase cells and the other types of cells sum up to less than 1,000. Obviously, the data sets are critically imbalanced. To handle this problem, we down-sample the interphase cells greatly while keeping other three classes of cells. But there are still serious problems with unequal distribution of the training examples, e.g., there are only 100 prophase cells and 306 anaphase cells.

### Image Processing and Feature Generation

During cell phase identification, it is critical to separate nuclei from the background. Nuclei are bright objects protruding out from a relatively uniform dark background. Digital images usually require pre-processing to remove noise, discard undesirable features, and correct illumination artifacts. In a sequence of cell images, our pre-processing procedure includes four steps: image enhancement, adaptive shareholding, morphological filtering, and distance transformation [1-4,11]. Although the adaptive threshold can segment all the cells from the background effectively, it cannot separate touching nuclei clusters. To solve this problem, our system utilizes a watershed algorithm. Traditional watershed segmentation, however, will lead to over-segmentation. Thus, a hybrid fragment merging approach that combines the roughness score and Probability Distribution Function (PDF) score of each cell is used [1-4,25-27]. This algorithm can effectively segment separated nuclei and most of the touching ones. The dynamic behaviors of cell nuclei are tracked by distance and size. After tracking, the performance of fragment merging is improved by the contextual information. The revised segmentation results are then used to reinforce the tracking performance.

After obtaining the segmented nuclei, feature vectors that each contains 145 features are generated to represent the cells. They compose of twelve general image features about shape, size, and intensity (max intensity, min intensity, deviation of gray level, average intensity, length of long axis, length of short axis, long axis/short axis, area, perimeter) [11]; 14 Haralick co-occurrence textural features [6,28]; 49 Zernike moment features [6,29], and 70 features generated by Gabor transformation [2,30].

### Parameters

To determine the dimensionality of the lower space for feature extraction algorithms, we vary the ratio of the energy preserved by feature extraction algorithms from 80% to 95% and compare the performance of different classifiers. Our results show that when the reduced dimensionality is 15, almost all the classifiers reach their best performance. Therefore, the reduced dimensionality for feature extraction algorithms is estimated as 15, with which 90% of the energy is preserved by PCA. With the FS algorithms, 10, 20, 30, 40, and 50 features are selected to compare the performance. We reduce the dimensions to 20 for all FS approaches. Both linear kernel and RBF kernel are used for the SVM classifier. The genetic algorithm is used in feature selection, and the parameters are as follows: populations size of 200, maximum generation size of 200, the portion of crossover is set to 0.5-th of the feature length, and the mutation rate is 0.3. One of the 200 populations is initialized with t-test feature selection method. The best performance of KNN is achieved by selecting K = 7 for cell phase identification. A BPNN [8] with a single hidden layer of 20 nodes is used to classify the four classes of cell phases, and is trained with back propagation algorithm. The best performance of SVM is always achieved by linear kernel except for the case without feature reduction. Only linear FE approaches are used in this paper due to their efficiency in contrast to nonlinear ones [31]. Thus, as indicated in Table 1 and 2, the best performance of SVM using linear kernel and RBF kernel is reported.

**Table 1:** The precision of the combinations of various classifiers and feature reduction algorithms.

classifier	FR algorithm	Precision (confidence Interval) (at 90% confidence level)			
		
		class 1	class 2	class 3	class 4
CBMM	PCA	0.9129 (0.8824,0.9433)	**0.8683 (0.7723,0.9643)**	0.9412 (0.9055,0.9770)	0.8586 (0.8011,0.9160)
	LDA	0.9021 (0.8786,0.9758)	0.8614 (0.8010,0.9218)	0.9496 (0.9432,0.9756)	0.8536 (0.8172,0.8900)
	MMC	**0.9170 **(0.8850,0.9490)	0.8417 (0.7253,0.9581)	0.9354 (0.8973,0.9734)	0.8022 (0.7672,0.8371)
	SDAFS	0.8487 (0.8064,0.8910)	0.7967 (0.7023,0.8906)	0.9484 (0.9048,0.9920)	0.8700 (0.8244,0.9156)
	MIFS	0.7902 (0.7449,0.8357)	0.7633 (0.6735,0.8531)	0.9483 (0.9204,0.9761)	0.8783 (0.8208,0.9358)
	GA	0.8556 (0.823,0.8883)	0.7833 (0.6584,0.9083)	**0.9642 (0.9336,0.9947)**	**0.8881 (0.8422,0.9340)**

SVM	PCA	0.9200 (0.9022,0.9375)	0.7518 (0.7045,0.7991)	0.9088 (0.8896,0.9279)	0.8069 (0.7721,0.8417)
	LDA	**0.9969 (0.9911,1.0026**)	0 (0,0)	0.8840 (0.8487,0.9193)	0 (0,0)
	MMC	0.9216 (0.9119,0.9314)	0.6655 (0.5946,0.7363)	0.8960 (0.8724,0.9195)	0.7190 (0.6670,0.7681)
	SDAFS	0.9457 (0.9372,0.9542)	0.6018 (0.5409,0.6627)	**0.9214 (0.9059,0.9369)**	0.8048 (0.7501,0.8596)
	MIFS	0.9457 (0.9257,0.9656)	0.6454 (0.5438,0.7470)	0.9086 (0.8819,0.9353)	**0.8401 (0.7913,0.8888)**
	GA	0.9381 (0.9194,0.9568)	**0.7827 (0.7173,0.8481)**	0.9172 (0.8902,0.9441)	0.8396 (0.7930,0.8661)

KNN	PCA	0.9487 (0.9349,0.9625)	**0.6691 (0.5927,0.7455)**	0.9215 (0.8977,0.9454)	0.7588 (0.7167,0.8001)
	LDA	0.7313 (0.6975,0.7652)	0.2773 (0.2005,0.3541)	0.2144 (0.1767,0.2520)	0.0922 (0.0605,0.1238)
	MMC	0.9532 (0.9367,0.9699)	0.6327 (0.5570,0.7084)	0.9171 (0.8984,0.9360)	0.7387 (0.6764,0.8010)
	SDAFS	0.9487 (0.9393,0.9582)	0.5110 (0.4324,0.5895)	0.9150 (0.8831,0.9469)	0.7908 (0.7541,0.8275)
	MIFS	0.9532 (0.9376,0.9689)	0.5518 (0.4832,0.6204)	0.9087 (0.8932,0.9242)	**0.8040 (0.7732,0.8347)**
	GA	**0.9668 (0.9481,0.9856)**	0.6273 (0.5469,0.7077)	**0.9384 (0.9276,0.9492)**	0.7841 (0.7420,0.8262)

BPNN	PCA	**0.9004 (0.8707,0.9301)**	**0.6817 (0.5352,0.8281)**	0.8876 (0.8369,0.9383)	**0.8106 (0.7669,0.8543)**
	LDA	0.8929 (0.8513,0.9345)	0.0100 (0.000,0.0283)	0.7746 (0.6147,0.9345)	0.2644 (0.1050,0.4238)
	MMC	0.8960 (0.8758,0.9162)	0.4664 (0.2381,0.6947)	**0.8895 (0.8501,0.9289)**	0.5919 (0.4672,0.7167)
	SDAFS	0.7472 (0.5156,0.978)	0.3364 (0.1228,0.5499)	0.7279 (0.5048,0.9509)	0.7032 (0.5559,0.8506)
	MIFS	0.8351 (0.6638,1.000)	0.4882 (0.2847,0.6917)	0.7342 (0.5084,0.9599)	0.7687 (0.608,0.9294)
	GA	0.7872 (0.6192,0.9552)	0.3791 (0.1147,0.6435)	0.7234 (0.5007,0.9461)	0.7341 (0.5679,0.9003)

The reduced dimensionality for feature extraction algorithm is 15, while the dimensionality for feature selection is 20. The best performance combination for each class and each classifier is displayed in bold.

**Table 2:** The sensitivity of the combinations of various classifiers and feature reduction algorithms.

classifier	FR algorithm	Sensitivity (confidence Interval) (at 90% confidence level)			
		
		class 1	Class 2	class 3	class 4
CBMM	PCA	**0.9390 (0.9100,0.9680)**	0.7602 (0.6786,0.8419)	0.9526 (0.9196,0.9856)	0.8575 (0.8026,0.9125)
	LDA	0.9220 (0.8966,0.9773)	0.7567 (0.8096,0.9237)	0.9489 (0.9462,1.000)	**0.9460 (0.9101,0.9818)**
	MMC	0.9202 (0.8979,0.9425)	**0.7733 (0.6724,0.8742)**	0.9289 (0.9086,0.9491)	0.8445 (0.7774,0.9116)
	SDAFS	0.9203 (0.8733,0.9401)	0.5537 (0.3536,0.4756)	0.9588 (0.9367,0.9817)	0.8786 (0.8191,0.9381)
	MIFS	0.9067 (0.8733,0.9401)	0.4146 (0.3536,0.4756)	0.9592 (0.9367,0.9817)	0.8786 (0.8191,0.9381)
	GA	0.9234 (0.8869,0.9599)	0.5812 (0.4748,0.6877)	**0.9611 (0.9344,0.9878)**	0.8743 (0.8273,0.9213)

SVM	PCA	0.8790 (0.8542,0.9038)	0.8025 (0.7328,0.8722)	0.9192 (0.9067,0.9317)	0.8744 (0.8441,0.9047)
	LDA	0.6254 (0.6128,0.6380)	NaN	0.8411 (0.8185,0.8636)	NaN
	MMC	0.8658 (0.8376,0.8941)	0.7694 (0.7061,0.8238)	0.8919 (0.8608,0.9230)	0.8221 (0.7882,0.8560)
	SDAFS	0.8731 (0.8519,0.8943)	0.7767 (0.7043,0.8491)	0.9267 (0.9086,0.9447)	**0.8966 (0.8734,0.9200)**
	MIFS	0.8819 (0.8607,0.9030)	0.7814 (0.7113,0.8515)	0.9421 (0.9247,0.9594)	0.8899 (0.8640,0.9157)
	GA	**0.8936 (0.8727,0.9146)**	**0.8332 (0.7689,0.8975)**	**0.9439 (0.9263,0.9615)**	0.8852 (0.8480,0.9226)

KNN	PCA	0.8570 (0.8328,0.8811)	0.8880 (0.8273,0.9488)	**0.9365 (0.9215,0.9515)**	0.8753 (0.8467,0.9041)
	LDA	0.4596 (0.4455,0.4736)	0.1751 (0.1406,0.2096)	0.4189 (0.3650,0.4729)	03689 (0.2580,0.4800)
	MMC	0.8590 (0.8367,0.8812)	0.9139 (0.8692,0.9587)	0.9042 (0.8913,0.9171)	0.8848 (0.8378,0.9318)
	SDAFS	0.8556 (0.8389,0.8722)	0.7410 (0.6738,0.8082)	0.9308 (0.9088,0.9527)	0.8958 (0.8776,0.9139)
	MIFS	0.8533 (0.8368,0.8698)	0.8312 (0.7372,0.9251)	0.9314 (0.9159,0.9467)	0.9027 (0.8740,0.9314)
	GA	**0.8741 (0.8556,0.8926**)	**0.9557 (0.9121,0.9993)**	0.9198 (0.9017,0.9379)	**0.9223 (0.8890,0.9568)**

BPNN	PCA	0.8493 (0.7781,0.9204)	**0.7500 (0.6490,0.8510)**	**0.9321 (0.9056,0.9585)**	**0.8177 (0.7701,0.8652)**
	LDA	0.7046 (0.6461,0.7631)	NaN	NaN	NaN
	MMC	0.8277 (0.7852,0.8702)	NaN	0.8407 (0.7881,0.8932)	0.7505 (0.6789,0.8221)
	SDAFS	NaN	NaN	NaN	NaN
	MIFS	**0.8683 (0.8302,0.9064)**	NaN	NaN	0.7935 (0.6939,0.8932)
	GA	NaN	NaN	NaN	NaN

The reduced dimensionality for feature extraction algorithm is 15, while the dimensionality for feature selection is 20. The best performance combination for each class and each classifier is displayed in bold.

### Measurements

We use *precision *and *sensitivity *as the measurements for our experimental results. Suppose TP, FP, and FN stand for the number of true positive, false positive and false negative samples respectively after the completion of cell phase identification. Precision is defined as precision = TP/(TP+FP), and sensitivity is defined as sensitivity = TP/(TP+FN). In other words, precision is the portion of cells identified positively that are really positive. Sensitivity refers to the ability to identify positive cells correctly. We can calculate the precision and sensitivity for each class if we treat one class as positive and other classes as negative. The average precision and sensitivity of the four classes is used to indicate the overall performance. The ten-fold cross validation is used for testing the trained classifiers. To address the statistical significance of differences in classification result, the confidence intervals of classification accuracies are estimated to be at the 90% confidence level.

### Testing Results

We first reduce the data to 15 dimensional vector spaces with different FE approaches and 20 for all FS approaches. Table 1 and 2 shows the results of all four classes (class1-interphase, class2-prophase, class3-metaphase, and class4-anaphase). We describe the details of all four classes instead of only their average, since prophase and metaphase identifications are very important for drug discovery application [1-4,11].

According to Table 1 and 2, it can be observed that CBMM has the best overall performance regardless of which feature reduction algorithm is coupled with. PCA combined with the CBMM classifier outperforms other combinations. Although LDA achieves similar performance, its use is not recommended due to the singularity problem [18].

**Figure 2:**
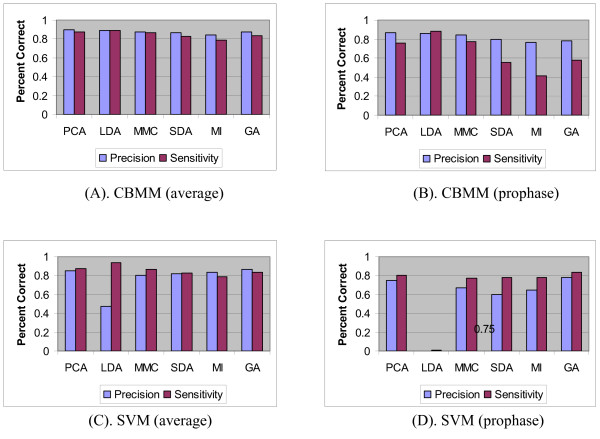
**Charts of average precision and sensitivity by CBMM and SVM**. Charts (A), (C) show the average precision and sensitivity of different classifiers; while (B), (D) show the precision and sensitivity of the prophase cell identified by different classifiers.

**Figure 3:**
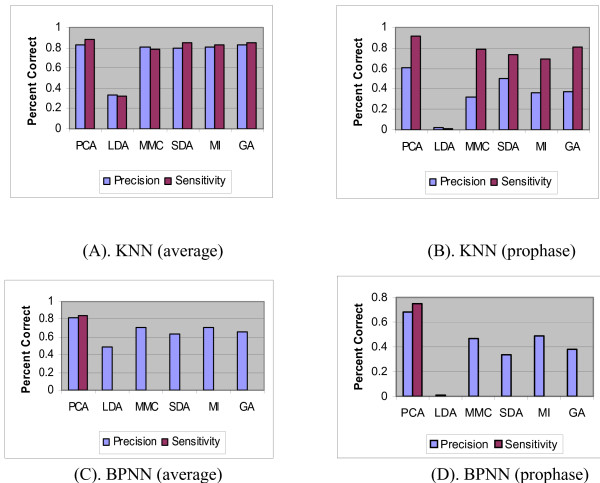
**Charts of average precision and sensitivity by KNN and BPNN**. Charts (A), (C) show the average precision and sensitivity of different classifiers; while (B), (D) show the precision and sensitivity of the prophase cell identified by different classifiers.

Since prophase is more important than the other three classes for drug screening, we show the specific measurement (precision and sensitivity) of prophase besides the average measurements of the four classes in Figure 2 and 3. CBMM outperforms SVM, BPNN, and KNN (Figure 2 and 3, (B), (D)) in classifying prophase. On the other hand, SDAFS has been reported to be the best among all the FS approaches [7], its performance when combined with CBMM is shown in Table 3 and 4. From Table 3 and 4, it can be observed that the optimal dimension for CBMM is 20 and its performance cannot be enhanced significantly by the increasing of the subspace dimension.

**Figure 4:**
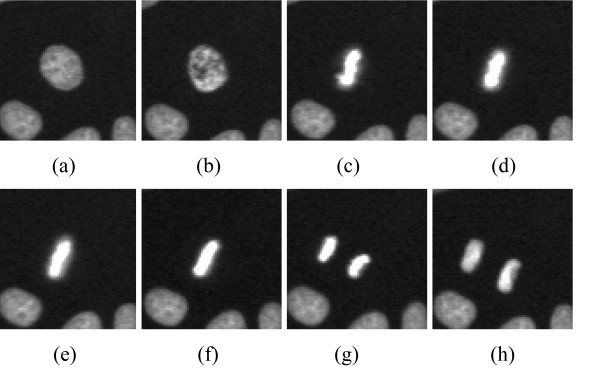
**Changes in the appearance of a nucleus during cell mitosis**. From (a) to (h) consecutive image subframes form a sequence showing nuclei size and shape changes during cell mitosis.

**Figure 5:**
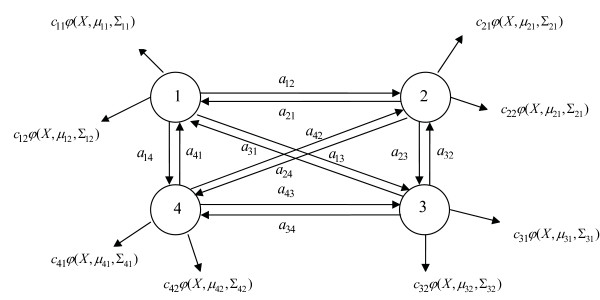
**An example of Continuous Gaussian Mixture Hidden Markov Model**. M = 4, R = 2, the prior probability of phases are *π*_*i*_, *i *= 1,2,3,4 which are ignored in this picture.

**Table 3:** The precision of SDAFS combined with CBMM when compared over a range of subspace dimensions.

	10-d	20-d	30-d	40-d	50-d
Class 1	0.8842 (0.7682,0.8958)	0.8320 (0.7682,0.8958)	0.7718 (0.7142,0.8293)	0.9933 (0.98113,1.000)	1 (1,1)
Class 2	0.785 (0.6918,0.8781)	0.7783 (0.6755,0.8812)	0.4917 (0.2869,0.6964)	0.01667 (0.0000,0.0472)	0 (0,0)
Class 3	0.8544 (0.7980,0.9108)	0.9529 (0.9143,0.9915)	0.7530 (0.6672,0.8388)	0.04 (0.0000,0.1056)	0 (0.0)
Class 4	0.7367 (0.6454,0.8281)	0.8729 (0.8217,0.9240)	0.8731 (0.8108,0.9353)	0.014286 (0,0.0404)	0 (0,0)

The dimensionality ranges from 10 to 50.

**Table 4:** The sensitivity of SDAFS combined with CBMM when compared over a range of subspace dimensions.

	10-d	20-d	30-d	40-d	50-d
Class 1	0.9047 (0.8773,0.9321)	0.9208 (0.8881,0.9536)	0.8061 (0.7394,0.8728)	0.4208 (0.3814,0.4602)	0.41667 (0.3860,0.4473)
Class 2	0.6505 (0.5384,0.7626)	0.5275 (0.5384,0.7626)	0.3582 (0.2327,0.4838)	NaN	NaN
Class 3	0.8944 (0.4603,0.5948)	0.9418 (0.9150,0.9687)	0.8810 (0.8282,0.9338)	NaN	NaN
Class 4	0.7052 (0.7797,0.9126)	0.8461 (0.7797,0.9126)	0.6908 (0.6119,0.7698)	NaN	NaN

The dimensionality ranges from 10 to 50.

These conclusions are drawn based on the preliminary analysis, which can serve as a guideline for future research. With the accumulation of new data, a more detailed and conclusive analysis will be presented in our future work.

### Implementation

The functions for PCA, LDA, MMC, SDAFS, and t-test are developed in Matlab 6.5. MIFS is adapted from previously reported source code [1]. The feature generation tools are adapted from [2,11] and [30], whereas the twelve general features are generated by Matlab. We use Libsvm [23] as the SVM classifier and implement the CBMM, BPNN, and KNN classifier in Matlab 6.5.

## Discussion

Our method can successively classify over 80% of the cell phases. Although such precision is acceptable for most biological applications, additional heuristic rules and online-training will improve the precision further. Since the cell cycle is confined by biological constraints, knowledge-driven heuristic rules can be applied to compensate for certain phase identification errors. For example, we are going to implement the following three biological rules to enhance the system performance:

• Phase progression rule: Once a cell enters a defined cell-cycle phase, it cannot go back to its previous phase.

• Phase timing rule: The time period that a cell stays in a phase also obey certain biological rules. Cells will usually stay in prophase no more than 45 minutes, metaphase for about 1 hour in untreated cell sequences, and anaphase under 1 hour. On the other hand, a cell can stay in interphase for more than 20 hours. In time-lapse sequences in a drug-treated cell population, certain cells can stay in metaphase for an even longer period of time.

• Phase continuation rule: Cells cannot skip the one cell cycle phase and enter next phase following the one it skipped, e.g., cells cannot jump from metaphase to interphase or from anaphase to metaphase.

It is worth noting that the problems with unequal distribution of training examples can be solved in a supervised framework while the unsupervised approach heavily depends on the distribution of training examples. For example, we may first down-sample the training sets with appropriate sampling method, then train the SVMs by assigning the training samples with different cost weights according to class size [23,24,32]. In the "weighted" SVM [23], the prediction accuracy of prophase can be increased by 10%~20% at the expense of slightly decrease of the classes with large samples using the reduced features. The weights for interphase, prophase, metaphase, and anaphase are 1, 10, 10, and 10 respectively.

## Conclusion

This paper proposes a new Context-Based Mixture Model for dealing with the time-series cell cycle sequence information, which outperforms other traditional classifiers in identifying prophase. The application of feature reduction techniques can effectively improve the prediction accuracy, whereas more features do not necessarily guarantee better performance.

## Methods

### Feature Reduction

From the Feature Reduction (FR) perspective, the traditional and the state-of-the-art dimensionality reduction methods can be generally classified into Feature Extraction (FE) and Feature Selection (FS) [15-21]. FE aims to project high dimensional data to a lower dimensional space by algebraic transformation according to certain criteria while FS identifies a subset of the most representative features according to pre-defined criteria, thus the features are ranked according to their individual predictive power. We compare certain commonly used feature reduction approaches below.

Series of cell divisions can be represented in lineage trees. For this study, we only use one of the daughter cells after each division. This results in only one cell being tracked in any given frame, thus a sequence of cell nuclei are tracked for all of the frames in the current experiment. In other words, a sequence of these cell nuclei is mathematically represented by a *n *× *d *matrix **X **∈ *R*^*n *× *d*^, where *d *is the number of features and *n *is the length of a cell sequence. Each cell nuclei is represented with a feature vector. **X**^*T *^is used to denote the transpose of matrix **X**. M sequences of cell nuclei (or M cell sequences) are denoted by a *mn *× *d *matrix X˜
 MathType@MTEF@5@5@+=feaafiart1ev1aaatCvAUfKttLearuWrP9MDH5MBPbIqV92AaeXatLxBI9gBaebbnrfifHhDYfgasaacH8akY=wiFfYdH8Gipec8Eeeu0xXdbba9frFj0=OqFfea0dXdd9vqai=hGuQ8kuc9pgc9s8qqaq=dirpe0xb9q8qiLsFr0=vr0=vr0dc8meaabaqaciaacaGaaeqabaqabeGadaaakeaaieqacuWFybawgaacaaaa@2DFA@ ∈ *R*^*mn *× *d*^. Each cell is denoted by a row vector **x**_*i*_, *i *= 1, 2,⋯, *mn*. Assume that these feature vectors belong to *c *different classes and the sample number of the *j*^*th *^class is *n*_*j*_, we use *c*_*j *_to represent class *j*, *j *= 1, 2,⋯, *c*. The mean vector of *c*_*j *_is mj=1nj∑xi∈cjxi
 MathType@MTEF@5@5@+=feaafiart1ev1aaatCvAUfKttLearuWrP9MDH5MBPbIqV92AaeXatLxBI9gBaebbnrfifHhDYfgasaacH8akY=wiFfYdH8Gipec8Eeeu0xXdbba9frFj0=OqFfea0dXdd9vqai=hGuQ8kuc9pgc9s8qqaq=dirpe0xb9q8qiLsFr0=vr0=vr0dc8meaabaqaciaacaGaaeqabaqabeGadaaakeaacqWGTbqBdaWgaaWcbaGaemOAaOgabeaakiabg2da9maaliaabaGaeGymaedabaGaemOBa42aaSbaaSqaaiabdQgaQbqabaaaaOWaaabuaeaaieqacqWF4baEdaWgaaWcbaGaemyAaKgabeaaaeaacqWG4baEdaWgaaadbaGaemyAaKgabeaaliabgIGiolabdogaJnaaBaaameaacqWGQbGAaeqaaaWcbeqdcqGHris5aaaa@4134@. The mean vector of all the cell nuclei is mall=1mn∑i=1mnxi
 MathType@MTEF@5@5@+=feaafiart1ev1aaatCvAUfKttLearuWrP9MDH5MBPbIqV92AaeXatLxBI9gBaebbnrfifHhDYfgasaacH8akY=wiFfYdH8Gipec8Eeeu0xXdbba9frFj0=OqFfea0dXdd9vqai=hGuQ8kuc9pgc9s8qqaq=dirpe0xb9q8qiLsFr0=vr0=vr0dc8meaabaqaciaacaGaaeqabaqabeGadaaakeaacqWGTbqBdaWgaaWcbaGaemyyaeMaemiBaWMaemiBaWgabeaakiabg2da9maaliaabaGaeGymaedabaGaemyBa0MaemOBa4gaamaaqadabaacbeGae8hEaG3aaSbaaSqaaiabdMgaPbqabaaabaGaemyAaKMaeyypa0JaeGymaedabaGaemyBa0MaemOBa4ganiabggHiLdaaaa@4238@. The feature reduction problem can be framed as the problem of finding a function *f *: *R*^*d *^→ *R*^*p *^according to an objective function *J*, where *p *is the dimension of data after the dimensionality reduction, so that an object **x**_*i *_∈ *R*^*d *^is transformed into **y**_*i *_= *f*(**x**_*i*_) ∈ *R*^*p*^.

### Classifiers

We select the established classifiers KNN [17], BPNN [8,17], and SVM [23,24] for comparison. In addition, the CBMM is also proposed to incorporate the contextual information.

### Context based Mixture Model Classifier

Figure 4 provides an example of cell mitosis process. The occurrence of phases in a sequence can be regarded as a stochastic process; hence, the cell sequence can be represented as a Markov chain where phases are in hidden states. The occurrence of the first phase in the sequence is characterized by the initial probability of the Markov chain. The occurrence of the other phases, which is given by the occurrence of its previous phase, is characterized by the transition probability. We calculate initial and transition probabilities for Markov chains with a set of training nuclei sequences. In addition, we assume each hidden state can generate a group of continuous visible states described by *R *Gaussian Mixtures. We optimize these Gaussian mixtures with the Expectation-Maximization (EM) algorithm. Those initial probabilities and the optimized Gaussian mixtures are regarded as a continuous Hidden Markov Model (HMM) [1,10] for the training sequences.

Mathematically, suppose a set of *N *training sequences (*χ*_1_, *χ*_2_,⋯*χ*_*N*_) is given and the phases of all the cell nuclei in these sequences are known. Each sequence is a cell nucleus in *T*_*l *_different frames χl={x1l,x2l,...xTll}
 MathType@MTEF@5@5@+=feaafiart1ev1aaatCvAUfKttLearuWrP9MDH5MBPbIqV92AaeXatLxBI9gBaebbnrfifHhDYfgasaacH8akY=wiFfYdH8Gipec8Eeeu0xXdbba9frFj0=OqFfea0dXdd9vqai=hGuQ8kuc9pgc9s8qqaq=dirpe0xb9q8qiLsFr0=vr0=vr0dc8meaabaqaciaacaGaaeqabaqabeGadaaakeaaiiGacqWFhpWydaWgaaWcbaGaemiBaWgabeaakiabg2da9iabcUha7Hqabiab+Hha4naaDaaaleaacqaIXaqmaeaacqWGSbaBaaGccqGGSaalcqGF4baEdaqhaaWcbaGaeGOmaidabaGaemiBaWgaaOGaeiilaWIaeiOla4IaeiOla4IaeiOla4Iae4hEaG3aa0baaSqaaiabdsfaunaaBaaameaacqWGSbaBaeqaaaWcbaGaemiBaWgaaOGaeiyFa0haaa@464D@, where each cell nucleus is denoted by a *p *dimensional vector xtl={xt1l,xt2l,...xtpl}
 MathType@MTEF@5@5@+=feaafiart1ev1aaatCvAUfKttLearuWrP9MDH5MBPbIqV92AaeXatLxBI9gBaebbnrfifHhDYfgasaacH8akY=wiFfYdH8Gipec8Eeeu0xXdbba9frFj0=OqFfea0dXdd9vqai=hGuQ8kuc9pgc9s8qqaq=dirpe0xb9q8qiLsFr0=vr0=vr0dc8meaabaqaciaacaGaaeqabaqabeGadaaakeaaieqacqWF4baEdaqhaaWcbaGaemiDaqhabaGaemiBaWgaaOGaeyypa0Jaei4EaSNaemiEaG3aa0baaSqaaiabdsha0jabigdaXaqaaiabdYgaSbaakiabcYcaSiabdIha4naaDaaaleaacqWG0baDcqaIYaGmaeaacqWGSbaBaaGccqGGSaalcqGGUaGlcqGGUaGlcqGGUaGlcqWG4baEdaqhaaWcbaGaemiDaqNaemiCaahabaGaemiBaWgaaOGaeiyFa0haaa@4A77@, *t *= 1,2,..., *T*. On the other hand, let *S *= {*s*_1_, *s*_2_,⋯, *s*_*M*_} be the set of *M *hidden states in Markov chain, we also consider the *N *training sequences as a group of *T*_*l*_*T*_*l *_length random variables, Θl={θ1l,θ2l,⋯,θTll}
 MathType@MTEF@5@5@+=feaafiart1ev1aaatCvAUfKttLearuWrP9MDH5MBPbIqV92AaeXatLxBI9gBaebbnrfifHhDYfgasaacH8akY=wiFfYdH8Gipec8Eeeu0xXdbba9frFj0=OqFfea0dXdd9vqai=hGuQ8kuc9pgc9s8qqaq=dirpe0xb9q8qiLsFr0=vr0=vr0dc8meaabaqaciaacaGaaeqabaqabeGadaaakeaacqqHyoqudaWgaaWcbaGaemiBaWgabeaakiabg2da9iabcUha7HGaciab=H7aXnaaDaaaleaacqaIXaqmaeaacqWGSbaBaaGccqGGSaalcqWF4oqCdaqhaaWcbaGaeGOmaidabaGaemiBaWgaaOGaeiilaWIaeS47IWKaeiilaWIae8hUde3aa0baaSqaaiabdsfaunaaBaaameaacqWGSbaBaeqaaaWcbaGaemiBaWgaaOGaeiyFa0haaa@46E1@. The *Sample Space *of these variables is *S*. By applying the Markov assumption (the state of an object at time *t *only depends on the state of it at time *t*-the conditional probabilities is: Pr⁡(θtl=sj|θt−1l=si,θt−2l=si2⋯θ1l=si1)=Pr⁡(θtl=sj|θt−1l=si)
 MathType@MTEF@5@5@+=feaafiart1ev1aaatCvAUfKttLearuWrP9MDH5MBPbIqV92AaeXatLxBI9gBaebbnrfifHhDYfgasaacH8akY=wiFfYdH8Gipec8Eeeu0xXdbba9frFj0=OqFfea0dXdd9vqai=hGuQ8kuc9pgc9s8qqaq=dirpe0xb9q8qiLsFr0=vr0=vr0dc8meaabaqaciaacaGaaeqabaqabeGadaaakeaacyGGqbaucqGGYbGCcqGGOaakiiGacqWF4oqCdaqhaaWcbaGaemiDaqhabaGaemiBaWgaaOGaeyypa0Jaem4Cam3aaSbaaSqaaiabdQgaQbqabaGccqGG8baFcqWF4oqCdaqhaaWcbaGaemiDaqNaeyOeI0IaeGymaedabaGaemiBaWgaaOGaeyypa0Jaem4Cam3aaSbaaSqaaiabdMgaPbqabaGccqGGSaalcqWF4oqCdaqhaaWcbaGaemiDaqNaeyOeI0IaeGOmaidabaGaemiBaWgaaOGaeyypa0Jaem4Cam3aaSbaaSqaaiabdMgaPnaaBaaameaacqaIYaGmaeqaaaWcbeaakiabl+Uimjab=H7aXnaaDaaaleaacqaIXaqmaeaacqWGSbaBaaGccqGH9aqpcqWGZbWCdaWgaaWcbaGaemyAaK2aaSbaaWqaaiabigdaXaqabaaaleqaaOGaeiykaKIaeyypa0JagiiuaaLaeiOCaiNaeiikaGIae8hUde3aa0baaSqaaiabdsha0bqaaiabdYgaSbaakiabg2da9iabdohaZnaaBaaaleaacqWGQbGAaeqaaOGaeiiFaWNae8hUde3aa0baaSqaaiabdsha0jabgkHiTiabigdaXaqaaiabdYgaSbaakiabg2da9iabdohaZnaaBaaaleaacqWGPbqAaeqaaOGaeiykaKcaaa@780A@, *i*, *j *= 1,2,⋯*M*. The trained model is represented by a group of parameters Λ = {∏, **A**, ***μ***_*kr*_, **Σ**_*kr*_, *c*_*kr*_, *k *= 1,2,⋯*M*, *r *= 1,2,⋯*R*}, where Π stands for the initial probability of each phase and **A **= {*a*_*ij*_} stands for the transition probability of Markov model.

For the Continuous Hidden Markov Model (CHMM), we assume each hidden state can generate *R *visible Gaussian mixtures *ϕ*(**x**, ***μ***_*kr*_, **Σ**_*kr*_), *k *= 1, 2,⋯, *M*, *r *= 1,2,⋯*R*, where **u**_*kr *_and **Σ**_*kr *_are means and covariance matrices of Gaussian mixtures respectively. In addition, we have a group of coefficients, *c*_*kr*_, to weight the Gaussian mixtures of each hidden state. Figure 5 provides an example of CHMM. The Gaussian mixtures of each phase can be initialized by Fuzzy K-means [17,19]. Eventually **u**_*kr *_and **Σ**_*kr *_are initialized based on the results of Fuzzy K-means. After initialization, the parameters **u**_*kr *_and **Σ**_*kr*_, *c*_*kr*_, *k *= 1,2,⋯*M*, *r *= 1,2,⋯*R *are optimized by EM algorithm iteratively.

The next issue is how to use this model to predict cell phases. According to traditional Continuous Gaussian Mixture HMM, the probability of a cell **x**_*t *_belonging to phase *s*_*m*_, i.e. *θ*_*t *_= *s*_*m*_, should only depend basically on **x**_*t *_and **x**_*t*-1_. Thus the probabilities we need for the categorization of cells could be denoted as *p*(*θ*_*t *_= *s*_*m*_|**x**_*t*_, **x**_*t*-1_). Based on the Bayesian formula, we rewrite them as:

p(θt=sm|xt,xt−1)=∑i=1Mp(θt=sm,θt−1=si|xt,xt−1)=∑i=1Mp(xt,xt−1|θt=sm,θt−1=si)p(θt=sm,θt−1=si)∑j=1M∑k=1Mp(xt,xt−1|θt=sj,θt−1=sk)p(θt=sj,θt−1=sk),     (1)
 MathType@MTEF@5@5@+=feaafiart1ev1aaatCvAUfKttLearuWrP9MDH5MBPbIqV92AaeXatLxBI9gBaebbnrfifHhDYfgasaacH8akY=wiFfYdH8Gipec8Eeeu0xXdbba9frFj0=OqFfea0dXdd9vqai=hGuQ8kuc9pgc9s8qqaq=dirpe0xb9q8qiLsFr0=vr0=vr0dc8meaabaqaciaacaGaaeqabaqabeGadaaakeaafaqaaeWabaaabaGaemiCaaNaeiikaGccciGae8hUde3aaSbaaSqaaiabdsha0bqabaGccqGH9aqpcqWGZbWCdaWgaaWcbaGaemyBa0gabeaakiabcYha8Hqabiab+Hha4naaBaaaleaacqWG0baDaeqaaOGaeiilaWIae4hEaG3aaSbaaSqaaiabdsha0jabgkHiTiabigdaXaqabaGccqGGPaqkaeaacqGH9aqpdaaeWbqaaiabdchaWjabcIcaOiab=H7aXnaaBaaaleaacqWG0baDaeqaaOGaeyypa0Jaem4Cam3aaSbaaSqaaiabd2gaTbqabaGccqGGSaalcqWF4oqCdaWgaaWcbaGaemiDaqNaeyOeI0IaeGymaedabeaakiabg2da9iabdohaZnaaBaaaleaacqWGPbqAaeqaaOGaeiiFaWNae4hEaG3aaSbaaSqaaiabdsha0bqabaGccqGGSaalcqGF4baEdaWgaaWcbaGaemiDaqNaeyOeI0IaeGymaedabeaakiabcMcaPaWcbaGaemyAaKMaeyypa0JaeGymaedabaGaemyta0eaniabggHiLdaakeaacqGH9aqpdaaeWbqaamaalaaabaGaemiCaaNaeiikaGIae4hEaG3aaSbaaSqaaiabdsha0bqabaGccqGGSaalcqGF4baEdaWgaaWcbaGaemiDaqNaeyOeI0IaeGymaedabeaakiabcYha8jab=H7aXnaaBaaaleaacqWG0baDaeqaaOGaeyypa0Jaem4Cam3aaSbaaSqaaiabd2gaTbqabaGccqGGSaalcqWF4oqCdaWgaaWcbaGaemiDaqNaeyOeI0IaeGymaedabeaakiabg2da9iabdohaZnaaBaaaleaacqWGPbqAaeqaaOGaeiykaKIaemiCaaNaeiikaGIae8hUde3aaSbaaSqaaiabdsha0bqabaGccqGH9aqpcqWGZbWCdaWgaaWcbaGaemyBa0gabeaakiabcYcaSiab=H7aXnaaBaaaleaacqWG0baDcqGHsislcqaIXaqmaeqaaOGaeyypa0Jaem4Cam3aaSbaaSqaaiabdMgaPbqabaGccqGGPaqkaeaadaaeWbqaamaaqahabaGaemiCaaNaeiikaGIae4hEaG3aaSbaaSqaaiabdsha0bqabaGccqGGSaalcqGF4baEdaWgaaWcbaGaemiDaqNaeyOeI0IaeGymaedabeaakiabcYha8jab=H7aXnaaBaaaleaacqWG0baDaeqaaOGaeyypa0Jaem4Cam3aaSbaaSqaaiabdQgaQbqabaGccqGGSaalcqWF4oqCdaWgaaWcbaGaemiDaqNaeyOeI0IaeGymaedabeaakiabg2da9iabdohaZnaaBaaaleaacqWGRbWAaeqaaOGaeiykaKIaemiCaaNaeiikaGIae8hUde3aaSbaaSqaaiabdsha0bqabaGccqGH9aqpcqWGZbWCdaWgaaWcbaGaemOAaOgabeaakiabcYcaSiab=H7aXnaaBaaaleaacqWG0baDcqGHsislcqaIXaqmaeqaaOGaeyypa0Jaem4Cam3aaSbaaSqaaiabdUgaRbqabaGccqGGPaqkaSqaaiabdUgaRjabg2da9iabigdaXaqaaiabd2eanbqdcqGHris5aaWcbaGaemOAaOMaeyypa0JaeGymaedabaGaemyta0eaniabggHiLdaaaaWcbaGaemyAaKMaeyypa0JaeGymaedabaGaemyta0eaniabggHiLdaaaOGaeiilaWIaaCzcaiaaxMaadaqadaqaaiabigdaXaGaayjkaiaawMcaaaaa@E9D0@

where *m *= 1,2,...*M*, *p*(*θ*_*t *_= *s*_*j*_, *θ*_*t*-1 _= *s*_*i*_) = *p*(*θ*_*t *_= *s*_*j*_|*θ*_*t*-1 _= *s*_*i*_)*p*(*θ*_*t*-1 _= *s*_*i*_) = *a*_*ij*_*π*_*i*_. The *p*(**x**_*t*_, **x**_*t*-1_|*θ*_*t *_= *s*_*j*_, *θ*_*t*-1 _= *s*_*i*_) means given *θ*_*t *_= *s*_*j *_and *θ*_*t*-1 _= *s*_*i*_, the probability of Gaussian mixtures *ϕ*(**x**, ***μ***_*jr*_, **Σ**_*jr*_) and *ϕ*(**x**, ***μ***_*kr*_, **Σ**_*kr*_), *r *= 1, 2,..., *R *can generate vectors **x**_*t *_and **x**_*t*-1_.

Traditional CHMM has utilized the information of the previous time point to predict the state of current time point. In our application, we know both the information of "left point" and "right point", since we have obtained the cell trace and the features of all cells. In contrast to the traditional CHMM introduced above, we propose to utilize the contextual information for cell phase identification, i.e., we propose to use Gaussian mixture models based on two-pixels. This model is called the Context Based Mixture Model Classifier.

p(θt=sm|xt,xt−1,xt+1)=∑i=1M∑j=1Mp(θt=sm,θt−1=si,θt+1=sj|xt,xt−1,xt+1)=∑i=1M∑j=1Mp(xt,xt−1,xt+1|θt=sm,θt−1=si,θt+1=sj)P(m,i,j)∑m=1M∑i=1M∑j=1Mp(xt,xt−1,xt+1|θt=sm,θt−1=si,θt+1=sj)P(m,i,j)     (2)
 MathType@MTEF@5@5@+=feaafiart1ev1aaatCvAUfKttLearuWrP9MDH5MBPbIqV92AaeXatLxBI9gBaebbnrfifHhDYfgasaacH8akY=wiFfYdH8Gipec8Eeeu0xXdbba9frFj0=OqFfea0dXdd9vqai=hGuQ8kuc9pgc9s8qqaq=dirpe0xb9q8qiLsFr0=vr0=vr0dc8meaabaqaciaacaGaaeqabaqabeGadaaakeaafaqaaeGabaaabaGaemiCaaNaeiikaGccciGae8hUde3aaSbaaSqaaiabdsha0bqabaGccqGH9aqpcqWGZbWCdaWgaaWcbaGaemyBa0gabeaakiabcYha8Hqabiab+Hha4naaBaaaleaacqWG0baDaeqaaOGaeiilaWIae4hEaG3aaSbaaSqaaiabdsha0jabgkHiTiabigdaXaqabaGccqGGSaalcqGF4baEdaWgaaWcbaGaemiDaqNaey4kaSIaeGymaedabeaakiabcMcaPiabg2da9maaqahabaWaaabCaeaacqWGWbaCcqGGOaakcqWF4oqCdaWgaaWcbaGaemiDaqhabeaakiabg2da9iabdohaZnaaBaaaleaacqWGTbqBaeqaaOGaeiilaWIae8hUde3aaSbaaSqaaiabdsha0jabgkHiTiabigdaXaqabaGccqGH9aqpcqWGZbWCdaWgaaWcbaGaemyAaKgabeaakiabcYcaSiab=H7aXnaaBaaaleaacqWG0baDcqGHRaWkcqaIXaqmaeqaaOGaeyypa0Jaem4Cam3aaSbaaSqaaiabdQgaQbqabaGccqGG8baFcqGF4baEdaWgaaWcbaGaemiDaqhabeaakiabcYcaSiab+Hha4naaBaaaleaacqWG0baDcqGHsislcqaIXaqmaeqaaOGaeiilaWIae4hEaG3aaSbaaSqaaiabdsha0jabgUcaRiabigdaXaqabaGccqGGPaqkaSqaaiabdQgaQjabg2da9iabigdaXaqaaiabd2eanbqdcqGHris5aaWcbaGaemyAaKMaeyypa0JaeGymaedabaGaemyta0eaniabggHiLdaakeaacqGH9aqpdaWcaaqaamaaqahabaWaaabCaeaacqWGWbaCcqGGOaakcqGF4baEdaWgaaWcbaGaemiDaqhabeaakiabcYcaSiab+Hha4naaBaaaleaacqWG0baDcqGHsislcqaIXaqmaeqaaOGaeiilaWIae4hEaG3aaSbaaSqaaiabdsha0jabgUcaRiabigdaXaqabaGccqGG8baFcqWF4oqCdaWgaaWcbaGaemiDaqhabeaakiabg2da9iabdohaZnaaBaaaleaacqWGTbqBaeqaaOGaeiilaWIae8hUde3aaSbaaSqaaiabdsha0jabgkHiTiabigdaXaqabaGccqGH9aqpcqWGZbWCdaWgaaWcbaGaemyAaKgabeaakiabcYcaSiab=H7aXnaaBaaaleaacqWG0baDcqGHRaWkcqaIXaqmaeqaaOGaeyypa0Jaem4Cam3aaSbaaSqaaiabdQgaQbqabaGccqGGPaqkcqWGqbaucqGGOaakcqWGTbqBcqGGSaalcqWGPbqAcqGGSaalcqWGQbGAcqGGPaqkaSqaaiabdQgaQjabg2da9iabigdaXaqaaiabd2eanbqdcqGHris5aaWcbaGaemyAaKMaeyypa0JaeGymaedabaGaemyta0eaniabggHiLdaakeaadaaeWbqaamaaqahabaWaaabCaeaacqWGWbaCcqGGOaakcqGF4baEdaWgaaWcbaGaemiDaqhabeaakiabcYcaSiab+Hha4naaBaaaleaacqWG0baDcqGHsislcqaIXaqmaeqaaOGaeiilaWIae4hEaG3aaSbaaSqaaiabdsha0jabgUcaRiabigdaXaqabaGccqGG8baFcqWF4oqCdaWgaaWcbaGaemiDaqhabeaakiabg2da9iabdohaZnaaBaaaleaacqWGTbqBaeqaaOGaeiilaWIae8hUde3aaSbaaSqaaiabdsha0jabgkHiTiabigdaXaqabaGccqGH9aqpcqWGZbWCdaWgaaWcbaGaemyAaKgabeaakiabcYcaSiab=H7aXnaaBaaaleaacqWG0baDcqGHRaWkcqaIXaqmaeqaaOGaeyypa0Jaem4Cam3aaSbaaSqaaiabdQgaQbqabaGccqGGPaqkcqWGqbaucqGGOaakcqWGTbqBcqGGSaalcqWGPbqAcqGGSaalcqWGQbGAcqGGPaqkaSqaaiabdQgaQjabg2da9iabigdaXaqaaiabd2eanbqdcqGHris5aaWcbaGaemyAaKMaeyypa0JaeGymaedabaGaemyta0eaniabggHiLdaaleaacqWGTbqBcqGH9aqpcqaIXaqmaeaacqWGnbqta0GaeyyeIuoaaaaaaOGaaCzcaiaaxMaadaqadaqaaiabikdaYaGaayjkaiaawMcaaaaa@1AC6@

where *p*(*m*, *i*, *j*) = *p*(*θ*_*t *_= *s*_*m*_, *θ*_*t*-1 _= *s*_*i*_, *θ*_*t*+1 _= *s*_*j*_) is the prior probability defined as:

P(θt=sm,θt−1=si,θt+1=sj)=#status of (i,j,m)∑i=1M∑j=1M∑m=1M#status of (i,j,m)     (3)
 MathType@MTEF@5@5@+=feaafiart1ev1aaatCvAUfKttLearuWrP9MDH5MBPbIqV92AaeXatLxBI9gBaebbnrfifHhDYfgasaacH8akY=wiFfYdH8Gipec8Eeeu0xXdbba9frFj0=OqFfea0dXdd9vqai=hGuQ8kuc9pgc9s8qqaq=dirpe0xb9q8qiLsFr0=vr0=vr0dc8meaabaqaciaacaGaaeqabaqabeGadaaakeaacqWGqbaucqGGOaakiiGacqWF4oqCdaWgaaWcbaGaemiDaqhabeaakiabg2da9iabdohaZnaaBaaaleaacqWGTbqBaeqaaOGaeiilaWIae8hUde3aaSbaaSqaaiabdsha0jabgkHiTiabigdaXaqabaGccqGH9aqpcqWGZbWCdaWgaaWcbaGaemyAaKgabeaakiabcYcaSiab=H7aXnaaBaaaleaacqWG0baDcqGHRaWkcqaIXaqmaeqaaOGaeyypa0Jaem4Cam3aaSbaaSqaaiabdQgaQbqabaGccqGGPaqkcqGH9aqpdaWcaaqaaiabcocaJiabdohaZjabdsha0jabdggaHjabdsha0jabdwha1jabdohaZjabbccaGiabd+gaVjabdAgaMjabbccaGiabcIcaOiabdMgaPjabcYcaSiabdQgaQjabcYcaSiabd2gaTjabcMcaPaqaamaaqahabaWaaabCaeaadaaeWbqaaiabcocaJiabdohaZjabdsha0jabdggaHjabdsha0jabdwha1jabdohaZjabbccaGiabd+gaVjabdAgaMjabbccaGiabcIcaOiabdMgaPjabcYcaSiabdQgaQjabcYcaSiabd2gaTjabcMcaPaWcbaGaemyBa0Maeyypa0JaeGymaedabaGaemyta0eaniabggHiLdaaleaacqWGQbGAcqGH9aqpcqaIXaqmaeaacqWGnbqta0GaeyyeIuoaaSqaaiabdMgaPjabg2da9iabigdaXaqaaiabd2eanbqdcqGHris5aaaakiaaxMaacaWLjaWaaeWaaeaacqaIZaWmaiaawIcacaGLPaaaaaa@8E79@

Since the denominator in (2) is the same for each class, in implementation we can neglect this term, i.e.,p(θt=sm|xt,xt−1,xt+1)∝∑i=1M∑j=1Mp(xt,xt−1,xt+1|θt=sm,θt−1=si,θt+1=sj)P(m,i,j)
 MathType@MTEF@5@5@+=feaafiart1ev1aaatCvAUfKttLearuWrP9MDH5MBPbIqV92AaeXatLxBI9gBaebbnrfifHhDYfgasaacH8akY=wiFfYdH8Gipec8Eeeu0xXdbba9frFj0=OqFfea0dXdd9vqai=hGuQ8kuc9pgc9s8qqaq=dirpe0xb9q8qiLsFr0=vr0=vr0dc8meaabaqaciaacaGaaeqabaqabeGadaaakeaacqWGWbaCcqGGOaakiiGacqWF4oqCdaWgaaWcbaGaemiDaqhabeaakiabg2da9iabdohaZnaaBaaaleaacqWGTbqBaeqaaOGaeiiFaWNaeCiEaG3aaSbaaSqaaiabdsha0bqabaGccqGGSaalcqWH4baEdaWgaaWcbaGaemiDaqNaeyOeI0IaeGymaedabeaakiabcYcaSiabhIha4naaBaaaleaacqWG0baDcqGHRaWkcqaIXaqmaeqaaOGaeiykaKIaeyyhIu7aaabCaeaadaaeWbqaaiabdchaWjabcIcaOiabhIha4naaBaaaleaacqWG0baDaeqaaOGaeiilaWIaeCiEaG3aaSbaaSqaaiabdsha0jabgkHiTiabigdaXaqabaGccqGGSaalcqWH4baEdaWgaaWcbaGaemiDaqNaey4kaSIaeGymaedabeaakiabcYha8jab=H7aXnaaBaaaleaacqWG0baDaeqaaOGaeyypa0Jaem4Cam3aaSbaaSqaaiabd2gaTbqabaGccqGGSaalcqWF4oqCdaWgaaWcbaGaemiDaqNaeyOeI0IaeGymaedabeaakiabg2da9iabdohaZnaaBaaaleaacqWGPbqAaeqaaOGaeiilaWIae8hUde3aaSbaaSqaaiabdsha0jabgUcaRiabigdaXaqabaGccqGH9aqpcqWGZbWCdaWgaaWcbaGaemOAaOgabeaakiabcMcaPaWcbaGaemOAaOMaeyypa0JaeGymaedabaGaemyta0eaniabggHiLdaaleaacqWGPbqAcqGH9aqpcqaIXaqmaeaacqWGnbqta0GaeyyeIuoakiabdcfaqjabcIcaOiabd2gaTjabcYcaSiabdMgaPjabcYcaSiabdQgaQjabcMcaPaaa@8E4B@.

To estimate *p*(**x**_*t*_, **x**_*t*-1_, **x**_*t*+1_|*θ*_*t *_= *s*_*m*_, *θ*_*t*-1 _= *s*_*i*_, *θ*_*t*+1 _= *s*_*j*_), the simplest way is to assume that the phase of three states are independent, then it can be simply approximated by the product of the three items *p*(**x**_*t*_|*θ*_*t *_= *s*_*m*_), *p*(**x**_*t*-1_|*θ*_*t*-1 _= *s*_*i *_) and *p*(**x**_*t*+1_|*θ*_*t*+1 _= *s*_*j*_). This assumption is not always true in realistic applications. In this case, we assume that they are dependent. More complex models are trained than the standard continuous models described above. We collect the cells in status satisfying (*θ*_*t *_= *s*_*m*_, *θ*_*t*-1 _= *s*_*i*_, *θ*_*t*+1 _= *s*_*j*_). For example, if (*θ*_*t *_= 2, *θ*_*t*-1 _= 1, *θ*_*t*+1 _= 3), the interphase cells are collected if they lie between the interphase and anaphase to estimate *p*(**x**_*i*_, **x**_*t*-1_, **x**_*t*+1_|*θ*_*t *_= 2, *θ*_*t*-1 _= 1, *θ*_*t*+1 _= 3) using the EM algorithm. Then we have 4 × 4 × 4 = 64 hidden states altogether. The hidden states with less than five training samples are assigned zero prior probabilities. Each hidden state can generate one visible Gaussian mixture *ϕ*(**x**, ***μ***_*k*_, **Σ**_*k*_), *k *= 1,2,...,64, where ***μ***_*k *_and **Σ**_*k *_sare means and covariance matrices of Gaussian mixtures respectively. The parameters ***μ***_*k *_and **Σ**_*k *_are optimized by the EM algorithm iteratively. Then we get *p*(*θ*_*i *_= *s*_*m*_|**x**_*i*_, **x**_*t*-1_, **x**_*t*+1_), *m *= 1,..., *M*, and classify cell *X*_*t *_to phase *s*_m*_, such that

θt=sm* iff sm*=arg⁡max⁡m=1,2,⋯M{p(θt=sm|xt,xt−1,xt+1)}
 MathType@MTEF@5@5@+=feaafiart1ev1aaatCvAUfKttLearuWrP9MDH5MBPbIqV92AaeXatLxBI9gBaebbnrfifHhDYfgasaacH8akY=wiFfYdH8Gipec8Eeeu0xXdbba9frFj0=OqFfea0dXdd9vqai=hGuQ8kuc9pgc9s8qqaq=dirpe0xb9q8qiLsFr0=vr0=vr0dc8meaabaqaciaacaGaaeqabaqabeGadaaakeaaiiGacqWF4oqCdaWgaaWcbaGaemiDaqhabeaakiabg2da9iabdohaZnaaBaaaleaacqWGTbqBcqGGQaGkaeqaaOGaeeiiaaIaeeyAaKMaeeOzayMaeeOzayMaeeiiaaIaem4Cam3aaSbaaSqaaiabd2gaTjabcQcaQaqabaGccqGH9aqpdaWfqaqaaiGbcggaHjabckhaYjabcEgaNjGbc2gaTjabcggaHjabcIha4bWcbaGaemyBa0Maeyypa0JaeGymaeJaeiilaWIaeGOmaiJaeiilaWIaeS47IWKaemyta0eabeaakiabcUha7jabdchaWjabcIcaOiab=H7aXnaaBaaaleaacqWG0baDaeqaaOGaeyypa0Jaem4Cam3aaSbaaSqaaiabd2gaTbqabaGccqGG8baFcqWH4baEdaWgaaWcbaGaemiDaqhabeaakiabcYcaSiabhIha4naaBaaaleaacqWG0baDcqGHsislcqaIXaqmaeqaaOGaeiilaWIaeCiEaG3aaSbaaSqaaiabdsha0jabgUcaRiabigdaXaqabaGccqGGPaqkcqGG9bqFaaa@6EE0@

## Abbreviations

**HMM: **Hidden Markov Model; **CHMM**: Continuous Hidden Markov Model; **SVM: **Support Vector Machine; **NN**: Neural Networks; **BPNN**: Back-Propagation Neural Networks; **KNN: **K-Nearest Neighbor; **PCA: **Principal Component Analysis; **SDAFS: **Stepwise Discriminate Analysis based Feature Selection; **LDA: **Linear Discriminant Analysis; **MMC: **Maximum Margin Criterion; **MIFS: **Mutual Information Feature Selection; **GA: **Genetic Algorithm; **GAFS: **Genetic Algorithm based Feature Selection; **TFS: **T-test Feature Selection; **HMMs: **Hidden Markov Models; **CBMM: **Context Based Mixture Model; **FE: **Feature Extraction; **FS: **Feature Selection; **PDF: **Probability Distribution Function.

## Authors' contributions

MW andXZ played equal roles in investigating the proposed approach and conducting the experiments. RWK generated the images of Hela cell line. STCW directed the project and guided the research discussion. All authors have read and approved the final manuscript.
